# Possibilities of Proteomics Profiling in Predicting Dysfunction of the Cardiovascular System

**DOI:** 10.3389/fphys.2022.897694

**Published:** 2022-04-25

**Authors:** V. B. Rusanov, L. Kh. Pastushkova, I. M. Larina, O. I. Orlov

**Affiliations:** State Scientific Center of the Russian Federation Institute of Biomedical Problems of Russian Academy of Sciences, Moscow, Russia

**Keywords:** space medicine, cardiovascular proteomics, protein signaling molecules, cardiovascular events, physiological regulation

## Introduction

In our opinion, cardiovascular proteomics is the most perspective field of proteomic research in space and Earth medicine. Proteomics, being still a relatively new discipline of fundamental research, is becoming not only a methodology for screening potential biomarkers in various cells, tissues, and biological fluids but also a method for determining the targets of therapeutic agents (Huang et al., 2017). Widely, understanding how multiple protein species interact to carry out regulation is an important objective of cardiovascular research. Besides, the power of proteomics to simultaneously provide information on the panoply of expressed proteins has made it uniquely suitable for resolving complex signaling conundrums and revealing cardiovascular diseases (CVD). (Lam et al., 2016). When using proteomic analysis in the cardiovascular system (CVS), two main strategies are relevant (Mokou et al., 2017): obtaining information about molecular physiological mechanisms and identification of biomarkers that reflect the dynamics of adaptation processes.

The variety of CVS adaptive responses is ensured by the high variability of the metabolic circuit of regulation of this system since numerous molecular mechanisms are involved in this process and, first of all, in the regulation of heart rate. From a physiological point of view, heart rate variability (HRV) is an integral characteristic that reflects not only the adaptive response of the CVS (Thayer and Lane, 2007; Thayer et al., 2010; Ernst, 2017) but also of more complex neural networks (Thayer et al., 2012).

However, the relationships between HRV and metabolic parameters under conditions of space flight (SF) conditions and clinical studies are poorly understood.

## Proteomic Profiling in Space Flight, Ground Model Experiments, and Clinical Studies

In our studies, we tried to describe some proteomic markers of the human cardiovascular system in SF and in-ground model experiments.

We have analyzed (Pastushkova et al., 2019) the characteristics of the HRV which can be reflected in the urine proteome in cosmonauts with different initial types of CVS regulation. In this report, we revealed that the concentration of three proteins: alpha-1 of collagen subunit type VI (COL6A1), mucin-1 (MUC1), cadherin-13 (CDH13), hemisentin-1 (HMCN1), semenogelin-2 (SEMG2), SH3 domain-binding protein (SH3BGRL3), transthyretin (TTR) and serine proteases inhibitors (IPSP) significantly differ between groups of cosmonauts with predominance parasympathetic or sympathetic tone. In our point of view, this fact characterized different strategies of adaptation in cosmonauts with different types of the predominance of autonomic tone.

In our next report (Pastushkova et al., 2020), these differences were confirmed by the fact that some biochemical parameters, were unidirectionally changed with three proteins on the 1-st and 7-the days after SF. These sets were totally different in classified groups. In group of cosmonauts with a predominance of the sympathetic tone were changed unidirectionally COL6A1 with potassium, ferrum, alpha-1. MUC1 with amylase, urea, inorganic phosphate, glucose, alkaline phosphatase, ionized calcium. CDH13 with uric acid, ferrum, alpha-1, potassium. In group of cosmonauts with a predominance of the parasympathetic tone: direct bilirubin, potassium, total calcium with COL6A1. Direct bilirubin, potassium with MUC1 and total ferrum biding capacity, transferrin, glucose gamma, globulin transferase with CDH13.

We hypothesized that the concentration of these proteins and their different relationship with some biochemical parameters reflect, as we called this process, “adaptation price” which depends on the type of autonomic regulation.

In some cases, studies in ground model experiments may be more productive than studies in the SF. Under conditions of 120-days isolation, we studied the participation of collagens, which are proteins of the extracellular matrix (ECM) and participate in the modulation of the biomechanical characteristics of the CVS. Our next report (Rusanov et al., 2022) presents these results. We hypothesized that collagens may be a biomarker of modulating influences of regulatory mechanisms of the circulatory system and also be markers of the aging process.

In-ground model experiments with “dry immersion” we identified a set of proteins with consisted of cell adhesion molecule 4 (CADM4), immunoglobulin heavy alpha-1 (IGHA1), serotransferrin (TRFE), tyrosine-protein kinase receptor (UFOAXL), galectin-3-binding protein (Gal-3BP) and matrix remodeling-associated protein (8MXRA8) and reflects, in our opinion, autonomic regulation of the cardiovascular system (Rusanov et al., 2020a). Moreover, correspondence of the results of an assessment of the direction, as well as the response time of various circuits of blood circulation regulation, which reflect their reactivity as an indicator of the adaptive capabilities of the body, has been demonstrated. It is shown that the results of the assessment of directivity, as well as the response time of various regulatory circuits in CVS, differed in the time range (Rusanov et al., 2020b).

The use of proteomic approaches, tested in space medicine, in clinical practice in patients with CVD would make it possible to expand the understanding of the development of the pathological process, which can serve as the basis for developing an individual treatment strategy, since a number of studies have shown high variability between people for various molecular indications, and individual initial variability was low (Tebani et al., 2020).

In an earlier publication (Pastushkova et al., 2017), we have profiled the urine proteome of 18 healthy subjects and compared it with the urine proteome profile of 18 patients with ischemic heart disease accompanied by hypertension. Comparison of urine proteome of healthy people and patients with postinfarction cardiosclerosis revealed proteins specific for patients with cardiovascular disease. Thus, proteins vitronectin (VTN), syndecan-4 (SDC4), a histidine rich glycoprotein (HRG), endothelial protein C receptor (EPCR), colony stimulating factor (CSFs), cathepsin D and sekretogranin-1(CTSD) may be considered (GHGB) as potential markers for CVD.

## Conclusion

Further research in the direction of cardiovascular proteomics should concern the clinical and experimental verification of the hypotheses. For public health, research in cardiovascular proteomics would help identify possible candidate proteins for more effective treatment and diagnosis of CVD. Furthermore, in our opinion, the identification and profiling of personalized metabolic markers is the way to personalized medicine.

Undoubtedly, proteomic research in space medicine has one serious limitation. The concept of a biomarker was proposed in 2001 by the US National Institutes of Health and includes a characteristic that can be objectively measured and that can serve as an indicator of physiological and pathological biological processes (Hoefner, 2001). Biomarker needs to be verified in large cohorts. The cohort of cosmonauts and even volunteers participating in model ground experiments is too small for this. However, we can try to identify proteins associated with the cardiovascular system and describe the signaling pathways of these proteins for further study as potential markers of physiological or pathological processes in CVS.
